# An Apparatus for Spectral Emissivity Measurements of Thermal Control Materials at Low Temperatures

**DOI:** 10.3390/ma12071141

**Published:** 2019-04-08

**Authors:** Jiayu Ma, Yuzhi Zhang, Lingnan Wu, Haogeng Li, Lixin Song

**Affiliations:** 1 Key Laboratory of Inorganic Coating Materials CAS, Shanghai Institute of Ceramics, Chinese Academy of Sciences, Shanghai 200050, China; majy@shanghaitech.edu.cn (J.M.); wulingnan@mail.sic.ac.cn (L.W.); zydylhg@163.com (H.L.); lxsong@mail.sic.ac.cn (L.S.); 2 Center of Materials Science and Optoelectronics Engineering, University of Chinese Academy of Sciences, Beijing 100049, China; 3 School of Physical Science and Technology, ShanghaiTech University, Shanghai 201210, China

**Keywords:** spectral emissivity, radiation, vacuum, low temperature

## Abstract

Thermal control materials are employed to adjust the temperature of a spacecraft operating in deep space. The spectral emissivity is a crucial factor in evaluating the thermal radiative properties of such materials. An apparatus, composed of a Fourier transform infrared spectrometer (FTIR), a sample cooling chamber and a mechanical modulation system was demonstrated to measure low temperature infrared spectral emissivity under vacuum. The mechanical modulation system, which includes a chopper and a lock-in amplifier, is employed to reduce the interference of background radiation during measurements. The limitation of the Fourier transform frequency on the chopper frequency can be eliminated by setting the FTIR on step-scan mode. The apparatus is separated into two parts and evacuated by different pumps. In this study, a high quality emission spectrum of a sample is measured by the apparatus. The spectral emissivity of thermal control materials are obtained in the wavelength range of 8 to 14 μm at 173 and 213 K. The combined standard uncertainty of the apparatus is 3.30% at 213 K.

## 1. Introduction

Emissivity is an important property of thermal control materials which are applied in the aerospace industry, such as in satellites, space telescopes, spacecrafts and space stations. Thermal control materials adjust the temperature of the equipment and make sure it works properly at low temperatures [1,2]. Deep space is a vacuum, black and cool environment. Heat transfer can only be carried out by radiation. To regulate the temperature of the equipment, the emissivity of the thermal control materials plays a decisive role. Thus, the low temperature emissivity measurement of these materials is essential under vacuum.

There are three main methods to measure the emissivity of thermal control materials: Calorimetric measurement, direct measurement and indirect measurement based on reflectivity. The calorimetric measurement has heat leakages that cannot be ignored at low temperature. This method can only provide total emissivity, which does not give the emissivity at a specific wavelength. For indirect measurements, samples with thin multi-layers cannot be measured because of the multiple reflections. However, we can obtain the spectral emissivity of such materials for the purposes of researching the vibration state of the material in the infrared via the direct measurement method. As a result, this study employs the direct emissivity measurement [1].

The accuracy of the measured results is affected by background radiation for the direct measurements. According to Planck’s law, radiation is greatly influenced by surface temperature, with the intensity of radiation increasing with temperature. Since the radiation intensity of the background at room temperature is much stronger than that of a sample at low temperatures (<233 K), sample radiation is easily drowned out, resulting in difficulty in detection. This fact makes it hard to obtain the spectral emissivity of a sample at low temperatures. Fourier transform infrared spectrometer (FTIR) measurements are very fast in a broad spectral region. Such devices are widely used to measure spectral emissivity. Furthermore, additional information, such as gas emission, can be inferred from the emissivity measurements [3,4,5,6,7].

To minimize the effect of background radiation, Monte et al [8] and Adibekyan [9,10] et al.used a FTIR with all the parts in the optical path cooled by liquid nitrogen, while Zhang et al. [11] used a chopper to modulate the radiation at high temperatures. In this work, we combined a chopper and a FTIR on step-scan mode. While a chopper is able to modulate radiance by blocking it at a specific frequency, a lock-in amplifier demodulates radiation using phase-sensitive detection technology. Setting a FTIR to step-scan mode can eliminate the limit of Fourier transform frequency given by an interferometer on the chopper frequency. In this way, step-scan modes can remove the background radiation well and thereby reduce the consumption of liquid nitrogen. 

From previous research, we know that water and carbon dioxide in the atmosphere have strong selective absorption of mid-infrared radiation [4]. To eliminate the influence of water and carbon dioxide and simulate the environment where thermal control materials are employed—space—we needed to keep the whole optic path under vacuum. This work demonstrates a new apparatus to measure the spectral emissivity of thermal control materials at low temperatures, illustrating its working principle in detail. 

## 2. Experimental Setup

**Figure 1:**
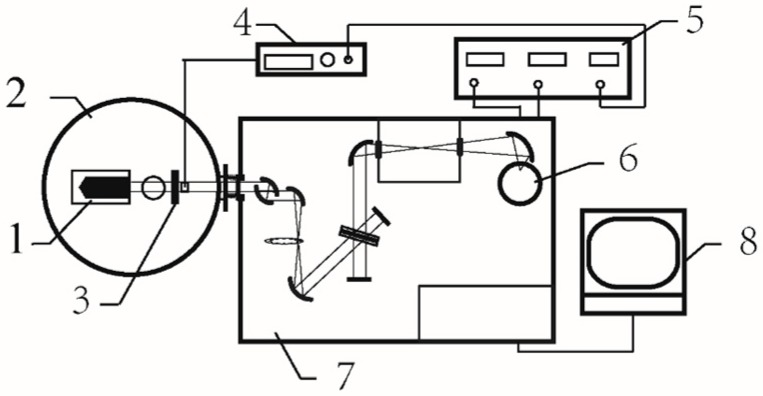
Schematic diagram of the measurement apparatus. 1—sample holder, 2—sample cooling chamber, 3—chopper wheel, 4—chopper controller, 5—lock-in amplifier, 6—mercury cadmium telluride detector, 7—Fourier transform infrared spectrometer, 8—computer.

The structure of the newly proposed apparatus, as shown in Figure 1, consists of a FTIR, a sample cooling chamber and a mechanical modulation system. There is an off-axis parabolic gold-coated mirror and a gold-coated plane mirror in the sample cooling chamber. The reflectivity of the two mirrors is higher than 0.96. The parabolic mirror is used to collect the radiation of the sample and transform the radiation into a parallel beam. The plane mirror is used to change the propagation direction of the parallel beam to make sure that the detector in the FTIR receives the radiation. The chopper is placed between the plane mirror and the input window of the FTIR. 

During measurements, the beam passed the chopper wheel in periodicity and was modulated in a specific frequency. Meanwhile, the chopper controller sent the reference signal—having the same frequency as the beam—to the reference channel of the lock-in amplifier [12,13,14]. The beam of radiation then passed the potassium bromide interferometer and was transformed into an interference light with the Fourier transform frequency. Finally, the interference light with the mechanical modulation frequency was detected by the mercury cadmium telluride (MCT) detector (InfraRed Associates, Stuart, FL, USA) with a ZnSe window cooled by liquid nitrogen. The MCT detector covered a spectral range of 2 to 20 μm and had a sensitivity of D*: >2 × 10^10^ cm·Hz ^1/2^·W^−1^ and SNR(Signal to Noise Ratio): 4542 at 4.5 μm.

Not only was the signal with modulation detected by the MCT detector, but also the background radiation at room temperature without modulation; both of these signals were sent to the signal channel of the lock-in amplifier. The lock-in amplifier amplified the signal and multiplied it by the reference signal using a phase-sensitive detector (PSD). The output of the PSD was then sent to the low pass filter of the lock-in amplifier. The signals with a different frequency from the reference signal were removed, resulting in a direct current (DC) signal containing only the radiation information from the sample. Finally, the DC signal was sent back to the FTIR [15,16]. The commercial software OPUS (7.8, Ettlingen, Germany) was used to help transform the interferogram of the DC signal into an emission spectrum.

To ensure the reliability of the FTIR in vacuum and reduce the loss of the radiation, a diamond window was used to separate the instrument into two parts. A double-sided flange and bellows were used to connect the spectrometer and the sample cooling chamber. The sample cooling chamber was able to be evacuated to 5 × 10^−3^ mbar by operating a molecular pump and a dry pump, while the FTIR could be evacuated to 1 mbar by applying a dry pump.

**Figure 2:**
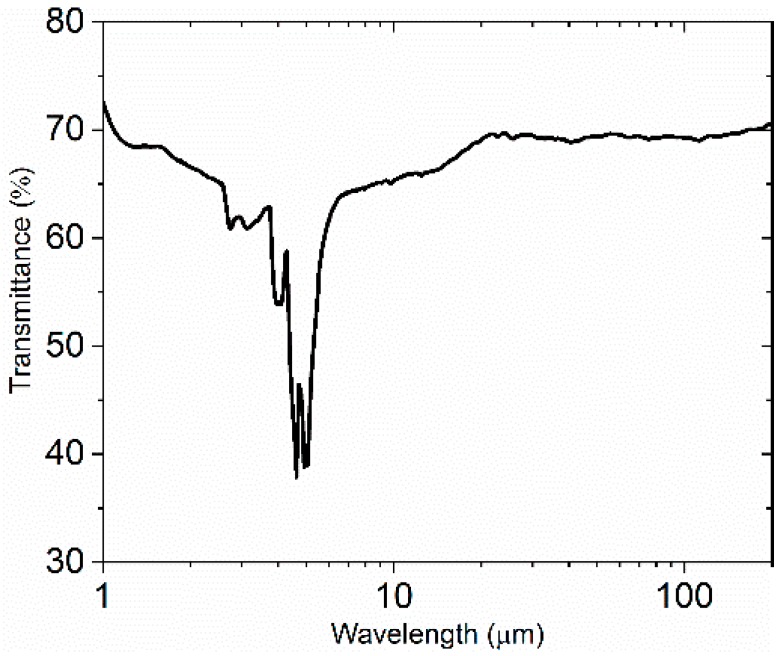
The transmittance of the diamond window.

We measured the transmittance of the diamond window in the wavelength range of 2 μm to 200 μm at room temperature in vacuum. As depicted in Figure 2, the transmittance of the diamond window increases from 64% to 70% over a wavelength increase of 8 to 11 μm, while the transmittance is fixed at about 70% over the wavelength range of 11 to 200 μm. The diamond window was a good selection for subsequent measurements at the far infrared. The transmittance curve only has sharp drops at 2.7 and 3.1 μm, and from 3.7 to 6.7 μm, which may be attributed to the strong absorption of all of the diamonds. The transmittance spectrum has no obvious drop at the wavelength range of 8 to 200 μm. This result indicates that the diamond window does not contain impurities [17].

**Figure 3:**
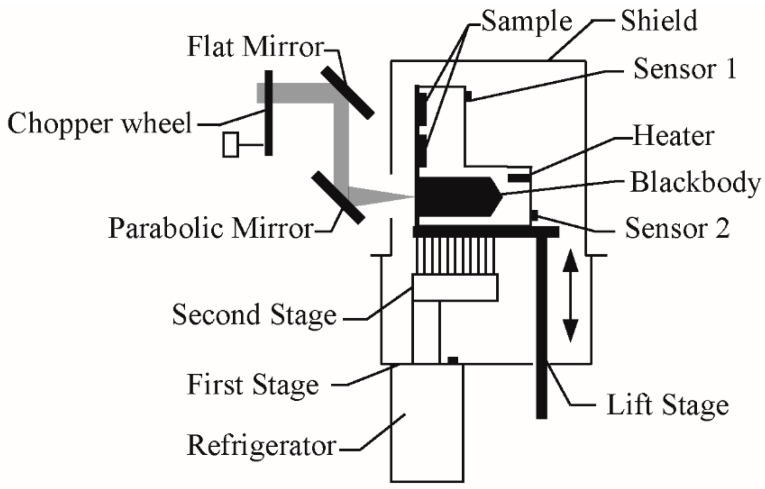
Schematic diagram of the sample cooling chamber.

Figure 3 shows the details of the sample cooling chamber. The sample holder, with a height of 150 cm, was made of copper possessing high thermal conductivity. Over the blackbody, there were two holes 24 mm in diameter and 5 mm in depth, used to place samples. The sample holder was an L-shape and could be divided into two parts: The vertical part with the two holes and the horizontal part where the blackbody was contained. The vertical part was thinner than the horizontal part. To remove the influence of the area difference, screws were used to fix 3 identical masks with an aperture before the samples and the blackbody. To keep good thermal contact, we fixed the sample to the hole with vacuum grease. A spring was set inside the hole to push the sample against the sample holder as much as possible.

Emissivity may be defined as the ratio of the radiation of a sample to the radiation of an ideal blackbody at the same temperature [11,18]. In fact, there was no ideal blackbody in this experiment. To get an accurate result, the emissivity of the blackbody should be as close to 1 as possible [1]. Thus, in this system, after considering factors such as the shape of the blackbody and the coating on it, the blackbody was designed into a cylindrical cavity with a conical bottom. In any case, the emissivity of the matte coating (Z306) on the blackbody was about 0.92 at room temperature. Additionally, we found that the emissivity of the blackbody was higher than 0.995, as calculated by the Gouffe method and the Monte Carlo method [19,20]. 

A refrigerator (ULCAC CRYOGENICS, Kanagawa, Japan) and a power adjustable heating resistor (SAKAGUCHI, Tokyo, Japan) were used to adjust the temperature of the sample holder. The refrigerator used was a closed cycle model and therefore able to cool down the sample holder without adding liquid helium, making it environmentally friendly and easy to operate. The heating resistor was put inside the sample holder and connected with the model 340 cryogenic temperature controller, operating through the use of a control loop to adjust the power and therefore the thermal resistance. Two silicon diode thermometers were fixed at the back of the sample holder. Sensor 1 measured the temperature of the vertical part, while sensor 2 determined the temperature of the horizontal part. To keep the temperature of the sample holder stable at the set point, the temperature controller adjusted the current flowing through the heating resistor, reducing the heating power when the temperature of sensor 2 was higher than the set value. 

In this system, the refrigerator had two tasks. The first task was used to cool down the stainless steel shield and keep its temperature lower than 55 K. The shield offered a stable, low temperature environment for the sample holder and was useful for keeping the temperature of the sample holder uniform. The parabolic mirror collected the radiation through an aperture in the shield to avoid interference between two samples. To minimize device radiation, the masks and the part of shield around the light aperture were covered with aluminum foil tap, which possesses low emissivity and is very stable at low temperature. The inner side of the shield was treated by sand slinging to avoid stray light being reflected to the sample.

The second task of the refrigerator was used to cool down the sample holder. The temperature of the second holder was lower than the first stage, and may be lower than 5 K. The sample holder and the second stage of the refrigerator were connected by braided copper straps and the ends of the straps were fixed on copper plates. To obtain optimized thermal contacts, we put indium tablets between the copper plate and the sample holder. By operating the refrigerator and the resistor in conjunction with one another, the temperature of the sample holder was extremely stable, with the drift of the temperature being controlled below 10 mK. 

The sample holder was installed on a lift stage with controller outside the sample cooling chamber. At the right side of the sample cooling chamber, there was a scale to show where the sample holder was. As a result, it was easy to adjust the position of the sample holder without opening the sample cooling chamber. A Teflon plat with poor thermal conductivity was set between the lift stage and the sample holder to avoid heat exchange.

Compared with traditional spectrometers, the FTIR can measure and record radiation in a broad spectral region quickly [3]. In this study, the FTIR employed was the Vertex 70V FTIR spectrometer from Bruker (Ettlingen, Germany). On normal scan mode, the moving mirror moves fast and continuously. The Fourier transform frequency $f_{\lambda }$ of the radiation transformed by the interferometer has the following relationship with the velocity of the moving mirror *v*:

(1) $$f_{\lambda }=2\nu /\lambda$$

where 2ν is the velocity of the optical path difference and λ is the wavelength of the radiation. For the wavelength 2.5–25 μm, the according $f_{\lambda }$ is 80 to 80 KHz [21,22]. In order to prevent interference between the Fourier transform frequency and the mechanical modulation frequency, the modulation frequency of the chopper had to be 10 times larger than the Fourier transform frequency. To solve this problem, we set the FTIR on step-scan mode. In this mode, the moving mirror is static at each step during measurement, so the velocity of the moving mirror, ν, is 0. Consequently, using Equation (1), the Fourier transform frequency $f_{\lambda }$ is found to be 0. As a result, the limitation of the Fourier transform frequency on choosing the frequency of the chopper is removed. 

## 3. Experimental Principle

Based on the definition of emissivity, the radiation of a sample surface received by the MCT detector can be written as follows:

(2) $$L_{\lambda ,eff}(T_{s})=\varepsilon_{\lambda ,s}(T_{s})L_{\lambda ,b}(T_{s})+[1-\varepsilon_{\lambda ,s}(T_{s})][L_{\lambda ,b}(T_{\mathrm{cham}})+\varepsilon_{\lambda ,sc}L_{\lambda ,b}(T_{\mathrm{sh}})]$$

where $\varepsilon_{\lambda ,s}(T_{s})$ is the sample emissivity at temperature $T_{s}$, $L_{\lambda ,b}(T_{s})$ is the blackbody radiation at the same temperature, and $1-\varepsilon_{\lambda ,s}(T_{s})$ is the reflectivity of the sample. Since the sample cooling chamber can be regarded as a blackbody, we use $L_{\lambda ,b}(T_{\mathrm{cham}})$ to express the radiation of the chamber at room temperature. $\varepsilon_{\lambda ,b}(T_{\mathrm{sh}})$ is the emissivity of the shield faced to the sample while $L_{\lambda ,b}(T_{\mathrm{sh}})$ is the emissivity of a blackbody at the shield temperature.

**Figure 4:**
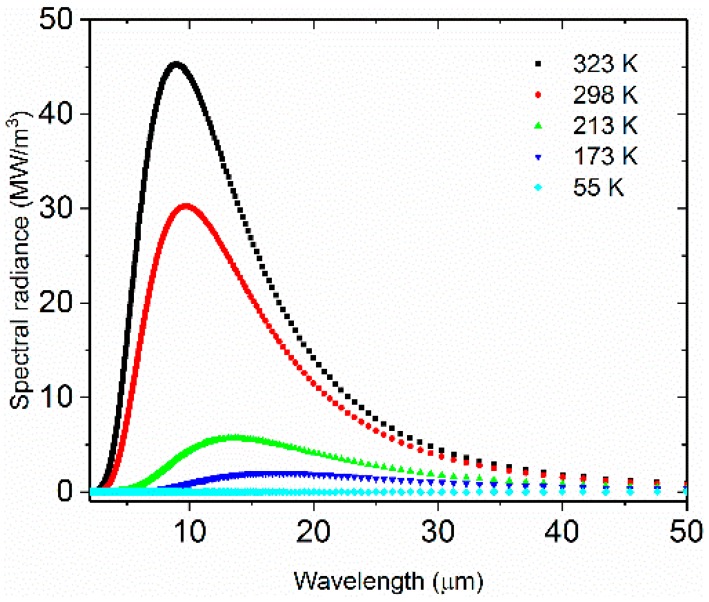
Spectral radiance of blackbody at 55, 298 and 323 K.

Using Planck’s law, we calculated the theoretical radiation of the blackbody at 55 K, 173 K, 213 K, 298 K and 323 K. As shown in Figure 4, the results indicated that the blackbody radiation at 55K is much smaller compared to the radiation at 298 K from 2 μm to 50 μm. Thus, the shield radiation at temperatures lower than 55 K may be considered negligible. This implies that *L_λ_*_,*b*_(*T*_cham_) + *ε_λ_*_,sh_*L_λ_*_,*b*_(*T*_sh_) ≈ *L_λ_*_,*b*_(*T*_cham_). When the sample temperature is 323 K, the radiation of the sample cooling chamber at 298 K is only slightly smaller than the sample, which will lead to an error during the emissivity measurement. The sample radiation at 173 K and 213 K are much smaller than the background radiation and can therefore be drowned out easily. We cannot obtain the emission spectrum of the sample without removing the influence of the background radiation.

When the chopper wheel blocks the light path, the MCT detector gets the radiation of the blade that is painted with a high emissivity (>0.95) coating and can therefore be treated as a blackbody as well.

The difference of the effective spectral radiance between the sample and the chopper wheel is reflected by the electrical signals of the MCT detector and may be written as:

(3) $$D_{\lambda ,s}=K\varepsilon_{\lambda ,s}(T_{s})[L_{\lambda ,b}(T_{\mathrm{cham}})-L_{\lambda ,b}(T_{s})]$$

where K is the apparatus function. Accordingly, the following formula shows the difference between the blackbody and the chopper wheel:

(4) $$D_{\lambda ,b}=K\varepsilon_{\lambda ,b}(T_{b})[L_{\lambda ,b}(T_{\mathrm{cham}})-L_{\lambda ,b}(T_{b})]$$

where *ε_λ,b_*(*T_b_*) is the spectral emissivity of the blackbody. Combining Equations (3) and (4), we obtain the following formula:

(5) $$\varepsilon_{\lambda ,s}(T_{s})=\varepsilon_{\lambda ,b}(T_{b})\frac{D_{\lambda ,s}[L_{\lambda ,b}(T_{\mathrm{cham}})-L_{\lambda ,b}(T_{b})]}{D_{\lambda ,b}[L_{\lambda ,b}(T_{\mathrm{cham}})-L_{\lambda ,b}(T_{s})]}$$

It is, in fact, very difficult to ensure that the sample and the blackbody are at the same temperature. In our system, we adjusted the blackbody temperature and the sample temperature at the sample time. In spite of using a material with a good thermal conductivity, there was still a temperature difference in different parts of the sample holder because of its large size. Specifically, we believe that the sample temperature is the same as the vertical part of the holder and the blackbody temperature is the same as the horizontal part. The temperature difference between the vertical part and horizontal part is 0.4 K when the temperature of the horizontal part is 50 K. If we assume *T_s_* = *T_b_* in Equation (5), then the equation can be quickly reduced to:

(6) $$\varepsilon_{\lambda ,s}(T_{s})=\varepsilon_{\lambda ,b}(T_{b})\frac{D_{\lambda ,s}}{D_{\lambda ,b}}$$

The emissivity of the sample $\varepsilon_{\lambda ,s}(T_{s})$ at temperature *T_s_*, is only determined by the emissivity of the blackbody *ε*_*λ*,*b*_ (*T_b_*) at temperature *T_b_*, and the radio of the electrical signals of the sample to the blackbody from the MCT detector [11]. Theoretically, the emissivity of the blackbody is exactly equal to 1. In Equation (6), the emissivity of the sample can be given by:

(7) $$\varepsilon_{\lambda ,s}(T_{s})=\frac{D_{\lambda ,s}}{D_{\lambda ,b}}$$

In the measurement, there is a temperature difference between the sample and the blackbody. To reduce the error caused by the temperature difference, we introduce a term $\gamma_{\lambda ,b}(T_{s})$. The term $\gamma_{\lambda ,b}(T_{s})$ is the ratio of the blackbody radiation at *T_b_* and *T_s_*. [11].

(8) $$\gamma_{\lambda ,b}=\frac{L_{\lambda ,b}(T_{b})}{L_{\lambda ,b}(T_{s})}$$

In the measurement, the emissivity of the sample at temperature *T_s_* is given by:

(9) $$\varepsilon_{\lambda ,meas}(T_{s})=\frac{L_{\lambda ,s}(T_{s})}{L_{\lambda ,b}(T_{b})}$$

where $\varepsilon_{\lambda ,meas}(T_{s})$ is the sample spectral emissivity calculated by measurements. After introducing the term, the spectral emissivity of the sample can be expressed as the ratio of the sample radiation and the blackbody radiation at the same temperature, and can be written as:

(10) $$\varepsilon_{\lambda ,\mathrm{corr}}(T_{s})=\gamma_{\lambda ,b}\frac{L_{\lambda ,s}(T_{s})}{L_{\lambda ,b}(T_{b})}=\frac{L_{\lambda ,s}(T_{s})}{L_{\lambda ,b}(T_{s})}$$

In Equation (10), $\varepsilon_{\lambda ,\mathrm{corr}}(T_{s})$ is the sample spectral emissivity after corrections. The error caused by the temperature difference between the sample and the blackbody is removed. Therefore, the spectral emissivity of the sample can be calculated by the following equation:

(11) $$\varepsilon_{\lambda ,\mathrm{corr}}(T_{s})=\gamma_{\lambda ,b}\frac{D_{\lambda ,s}}{D_{\lambda ,b}}$$

## 4. Procedure of Calibration

To verify that the system could remove the background radiation, we measured the emission spectrum of the stainless steel sample on normal scan mode without the chopper and on step-scan mode with the chopper in the wavelength range of 8 to 14 μm at 323 K in air. The sample was a round piece of plate, placed on the bottom hole of the sample holder. The apertures of the masks were 15 mm in diameter. To transmit the sample radiation as much as possible, the chopper wheel was rotated to the position where it did not block the passage of the radiation. The temperature of sensor 2 was set to 323 K to make sure that the sample radiation was sufficiently larger than the background radiation.

Compared with sample radiation at high temperature, the background radiation at room temperature is small and may be ignored. The emission spectrums of a sample and a blackbody could be obtained by a detector without chopping, and the results used to calculate sample emissivity. However, this method cannot work when the sample temperature is lower than the background temperature.

**Figure 5:**
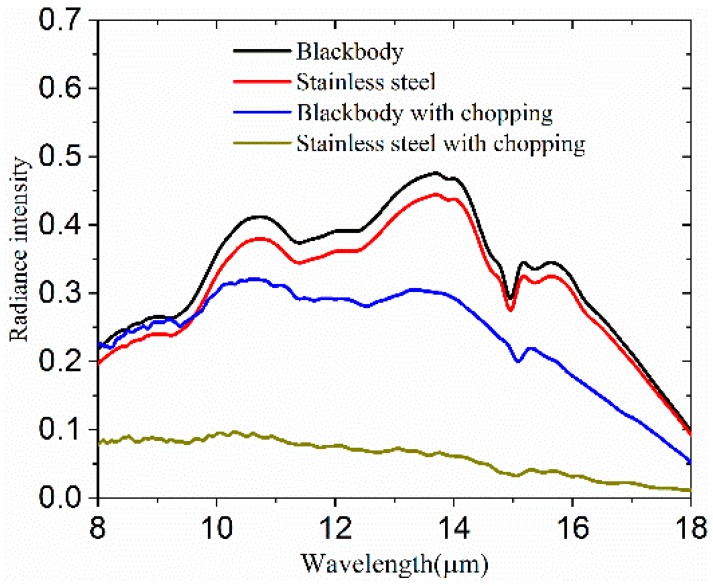
Spectral radiance spectrums of stainless steel and blackbody, with and without modulation, at 323 K.

In this study, a FTIR on normal scan mode was firstly used without chopping to detect the radiation of the stainless steel sample and the blackbody. Stainless steel is a typical low-emitting material, widely used in aerospace at low temperature. Theoretically, the emission spectrum of stainless steel should be vastly different to the emission spectrum of the blackbody at the same temperature. However, the two emission spectrums were found to be very similar, as shown in Figure 5. This is because both spectrums include the background radiation at room temperature. As shown in Figure 5, the intensity at 14 μm is larger than the corresponding value 10.5 μm. There is an obvious absorption peak in the wavelength range of 14 to 16 μm. If the emissivity of stainless steel was calculated according to these measurements, a great error would occur in the results.

Following these findings, the chopper was then turned on and the chopping frequency set to 330 Hz, while the FTIR was set to step-scan mode. As depicted in Figure 5, the difference between the spectral radiance of the stainless steel sample and the blackbody is very obvious. The radiance intensity at 14 μm is weaker than the value at 10.5 μm. The absorption peak has almost disappeared in the emission spectrum of stainless steel, and the radiance intensity of stainless steel is much smaller than the blackbody. The results indicate that our system is able to make a distinction between materials with different emissivity, removing background radiation in spectral radiance measurements. Furthermore, this method is easier and simpler as it does not require cooling down of the whole light path with liquid nitrogen in order to decrease the radiance intensity of the background. To check the stability of the instrument, we measured the emission spectrum of stainless steel five times at 173 K. The results show high reproducibility of the measurement procedure. We also measured the blackbody emission spectrum with different chopping frequencies, with the results showing no difference. Thus, the chopping frequency does not appear to be a factor which affects the measurements of the emission spectral.

## 5. Results

**Figure 6:**
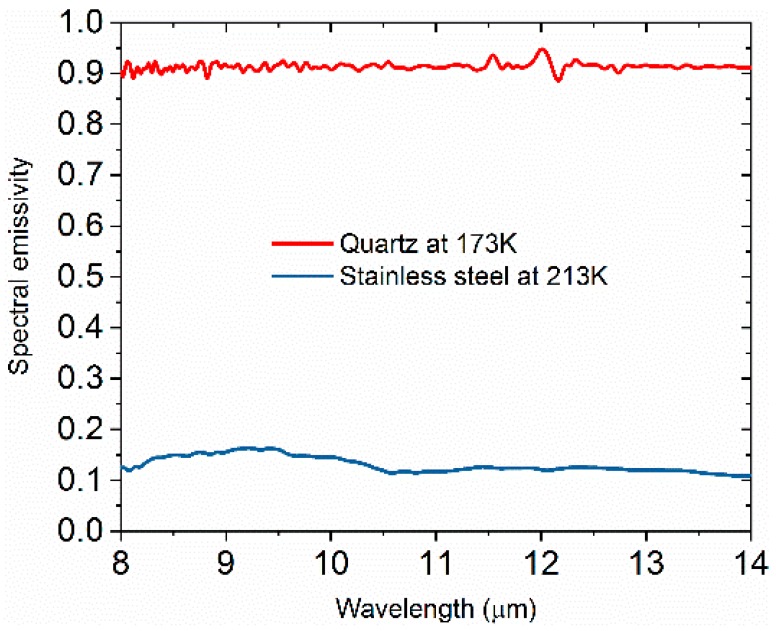
Spectral emissivity of quartz and stainless steel at 173 and 213 K, respectively.

From the results above, we know that the mechanical modulation and the interferometer modulation can work well with each other in our system. Thus, we started to carry out experiments at low temperatures under vacuum. In the experiments, we obtained the spectral emissivity of quartz and stainless steel. The MCT detector was cooled down by liquid nitrogen to 77 K. To make sure the MCT detector was sensitive to the sample radiation, it was important that the temperature of the sample was higher than the MCT detector. Consequently, we set the measurement temperature of the quartz sample to 173 K. Quartz is an opaque material in the infrared band and has high emissivity at room temperature. During measurement, quartz was fixed at the top hole of the sample holder, while stainless steel was fixed at the bottom hole. The results of the measurements are shown in Figure 6. The spectral emissivity of the quartz sample was found to be about 0.9 in the wavelength range of 8–14 μm, and hardly varied with the wavelength. As seen in Figure 6, the spectral emissivity of the quartz had a fluctuation in the wavelength range of 8 to 10 μm. The spectral emissivity of the stainless steel sample ranged from 0.11 to 0.15. The spectral emissivity of the stainless steel decreased as the wavelength increased. In Figure 6, a peak is observed on the spectral emissivity curve for stainless steel in the wavelength range of 8 μm to 10.5 μm, similar to the findings reported by Xu et al. [23]. In the case of small temperature differences, the emissivity value of the stainless steel sample in the range 8–14 μm was close to the results reported by Makino et al. [24]. These results support the notion that this apparatus is able measure the spectral emissivity of materials with different emissivity.

During experimentation, the size-of-source effect was eliminated by using the same masks. The nonlinearity of the FTIR was removed with the development of technology. The main sources of uncertainty in spectral emissivity are sample temperature, blackbody temperature, and blackbody emissivity, among others [11,25,26,27], which are considered in detail in this section. Table 1 shows the standard emissivity uncertainties of stainless steel at 213 K, at the wavelength of 10 and 12 μm.

Sample temperature and blackbody temperature were measured using two silicon diode thermometers (DT64-BO), with the uncertainty—provided by the manufacturer—evaluated as ±0.1 K. The refrigerator and heating resistor were used to regulate the temperature of the sample holder where the sample and blackbody were fixed on. When the drift of the sample temperature and the blackbody temperature were below 0.01 K, the temperature was considered stable. The emissivity of the blackbody used in this system was calculated to be 0.995 by the Gouffe method and the Monte Carlo method. The emissivity uncertainty of the stainless steel caused by the blackbody emissivity was 0.005. Because of the large size of the sample holder, the uncertainty of the temperature uniformity was 1 K, and was therefore the major source of uncertainty. In the future, the most important way to improve measurement accuracy would be to reduce the temperature difference between the sample and the blackbody. The combined standard uncertainty, calculated with the mentioned uncertainties, was found to be 3.3% and 2.8% at the wavelength of 10 and 12 μm, respectively.

## 6. Summary

In this paper, the experimental system and measurement principle for a low temperature spectral emissivity measurement under vacuum is presented. In this apparatus, the FTIR on step-scan mode can work well with a chopper and lock-in amplifier to remove the influence of background radiation effectively and easily. The equation, based on reasonable assumptions and simplifications, used to calculate spectral emissivity, is very concise. In the case of small temperature differences, the emissivity value of stainless steel in the range of 8–14 μm is close to results reported in the literature, confirming the rationality of the experimental setup and procedures. For low emissivity samples at 213 K, the combined standard uncertainty of the spectral emissivity was less than 3.3% in the wavelength range of 8 to 14 μm. This system provides a way to measure the spectral emissivity of materials, which can help to understand their thermal radiative properties.

## Figures and Tables

**Table 1:** Standard uncertainty of the emissivity of the stainless steel at 213 K.

Uncertainty Contribution	Type	∆x	∆ε (%)	
			10 μm, ε = 0.146	12 μm, ε = 0.121
Blackbody temperature	B	0.2 K	0.636	0.532
Stability of the blackbody temperature	A	0.01 K	0.032	0.027
Blackbody emissivity	B	0.005	0.005	0.005
Sample temperature	B	0.2 K	0.318	0.532
Stability of the sample temperature	A	0.01 K	0.032	0.027
Temperature uniformity	B	1 K	3.177	2.654
Total uncertainty in emissivity	–	–	3.302	2.759
